# Domestic Medicine

**Published:** 1871-01

**Authors:** 


### Domestic Medicine.

### USEFUL RECIPES FOR THE HOUSEHOLD.

DIABETES.—The worst forms of this distressing disease are cured simply by means of a milk diet. The patient should not be allowed to eat nor drink anything but skim-milk, in doses of a wine glass or gobulet full, every two or four hours, during the day or evening. This treatment is now adopted by the most scientific physicians of this country and England.

A REMEDY FOR SPASMS.—

Take of

Acitate of morphia, one grain;
Spirits of sal volatile,
Sulphuric ether, of each, one fld. oz;
Camphor Julep, four fld. oz.

Mix. It should be kept closely corked, in a cool place, and must be well shaken before use.

*Dose:*—A teaspoonful in a wine glass of cold water.

COUGH MIXTURE.—The following will be found to be an excellent cough mixture. Have your druggist prepare it for you and keep it constantly in the house, ready for use for any cough or cold which any member of your family is liable to have this winter.

Take:—

Syrup of Tolu,
Syrup of peru,
Syrup of sanguinaria,
Syrup of lobelia, of each, one ounce.
Tincture of wintergreen, one drachm.

Mix. *Dose:*—Half a teaspoonful three or four times daily, until the cough stops.

TO REDUCE A RUPTURE.—Place the patient in a hot, hip bath, and while there give him cupfull after cupfull of strong coffee, without sugar or milk. After he has drank all he can possibly swallow, dry him thoroughly, and place him in bed, upon his back, and elevate the foot of the bed two or three feet higher than his head. Then apply the hand, previously warmed in warm water, to the tumor and gradually press upon it until you hear a gurgling sound, accompanied with the disappearance of the tumor into the abdominal cavity.

This treatment will succeed nine times out of ten, in the most severe cases of rupture, in either sex.

TAPE WORM.—A perfectly safe prescription, and one that is very certain to destroy the worm, too, is to take a half pint of pumpkin seeds, pour one quart of water upon them, and allow them to simmer upon the stove, until evaporated to one pint. Then strain the tea from the seeds, and drink the pint at one dose, after fasting for twenty-four hours. Four hours after drinking the tea, take a large dose of castor oil. You will have the satisfaction of seeing the worm come away entire.

---

BEEF TEA.—This is one of the most nourishing, and at the same time easily digested food that can be administered to a convalescing or enfeebled patient. Very few nurses know how to make it properly, and unless it *is* properly made it is worse than nothing for a weak stomach. Take half a pound of fresh killed beef for every pint of tea desired, being careful to remove all fat, gristle and bone. Cut the meat into pieces about half an inch square, and set them to soak in a tea cup full of water—if the half pound is used—for six or eight hours. Then take the meat out, place it in a glass jar, pour a pint of water over it, set it in a kettle of water and boil it for three hours. Then take it out, strain it, and add the resulting liquor to that in which the meat had been previously soaked. Dry the particles of meat in the oven, chop and pound them perfectly fine, add them to the tea, and you have most a nourishing and digestible meal for any sick person.

---

DECAYED TEETH.—Very many diseases of the stomach, eyes, ears, brain and nervous system, are caused by a mouthful of decayed teeth. We have seen many diseases of the eye and stomach particularly, that we have been able to trace directly to this cause. No person can be healthful with their teeth in a decayed, ulcerating and stinking condition. It is not only an offence to the health, but an insult to every person who is compelled to stand near such an individual and inhale his offensive breath, as well as to look upon his filthy teeth. Either have your teeth renovated, or secure the service of a Dentist, and have them extracted. You will not only benefit your own health, but that also of every person who is compelled to come in contact with you.

---

SHOUDLER BRACES.—Do not wear them. They do not answer the purpose for which they are intended, but are an absolute harm. They perform the work that should be accomplished by the muscles of the breast and back, and by so doing weaken instead of strengthening them. By the use of dumb bells or the Indian clubs, you can easily overcome any disposition to "stoop shoulders," without the aid of worse than useless braces.

---

GLANDERS.—This fearful disease is contracted from the horse. It is contagious, so that persons working about animals afflicted with the disorder, should be careful not to have any of the matter come in contact with the eye, lips, nose, or any sore spot upon the face or hands. If such an accident should happen, innoculation would take place, which would subject the person to a most frightful disease that would certainly result in a painful death. We have seen the reports of several cases of glanders, in our medical exchanges, recently, one of which was that of a distinguised Russian Lady, who, in attempting to pick up her handkerchief, which had dropped near the curb stone, brought her face in contact with a horse's lip, which was suffering from glanders. She was taken violently ill the following day and suffered terribly with the disgusting disease for about two weeks, when she expired. Persons cannot be too careful when about horses suffering with glanders. The safest plan would be to destroy the animals, as they are dangerous to have about the premises and are rarely of any service after they have contracted the disease.

